# Hipertensão Arterial e Parâmetros Lipídicos, Glicídicos e de Adiposidade Associados em Adolescentes Escolares do Distrito Federal

**DOI:** 10.36660/abc.20201240

**Published:** 2022-01-11

**Authors:** Letícia Rocha Lima, Aline Bassetto Okamura, Kênia Mara Baiocchi de Carvalho, Eliane Said Dutra, Vivian Siqueira Santos Gonçalves

**Affiliations:** 1 Departamento de Nutrição Universidade de Brasília Brasília DF Brasil Departamento de Nutrição - Universidade de Brasília, Brasília, DF – Brasil; 2 Programa de Pós-graduação em Saúde Coletiva Universidade de Brasília Brasília DF Brasil Programa de Pós-graduação em Saúde Coletiva - Universidade de Brasília, Brasília, DF – Brasil; 3 Programa de Pós-graduação em Nutrição Humana Universidade de Brasília Brasília DF Brasil Programa de Pós-graduação em Nutrição Humana - Universidade de Brasília, Brasília, DF – Brasil

**Keywords:** Hipertensão, Adolescente, Adiposidade, Glicemia, Lipídeos

## Abstract

**Fundamento:**

A prevalência de hipertensão arterial sistêmica (HAS) e de outros distúrbios metabólicos tem aumentado em indivíduos jovens. Entretanto, não há estudos representativos sobre esse assunto com a população do Distrito Federal (DF).

**Objetivo:**

Estimar a prevalência de HAS e a sua associação com parâmetros lipídicos, glicídicos e de adiposidade em adolescentes do DF.

**Métodos:**

Trata-se de um estudo observacional transversal com participantes do Estudo de Riscos Cardiovasculares em Adolescentes (ERICA). Foram avaliados pressão arterial, glicemia sanguínea, hemoglobina glicada, insulina, modelo de avaliação da homeostase da resistência à insulina (HOMA-IR), triglicerídeos, colesterol total, lipoproteína de alta densidade, lipoproteína de baixa densidade, índice de massa corporal (IMC) e perímetro da cintura, além de variáveis econômicas, demográficas e de maturação sexual. A análise de dados foi feita no *software* Stata e foi dividida nas seguintes etapas: análises descritiva, bruta e ajustada. Considerou-se p < 0,05.

**Resultados:**

Foram incluídos 1.200 adolescentes com média de idade de 14,8 anos. A prevalência de HAS foi de 8% (intervalo de confiança de 95%: 6,3; 9,9). A maioria dos parâmetros se associou com a PA na análise bruta; na ajustada, os parâmetros glicídicos, lipídicos e de adiposidade mantiveram a associação, tendo IMC e HOMA-IR a maior magnitude na relação.

**Conclusão:**

O estudo revelou elevada prevalência de HAS em adolescentes do DF, e os níveis pressóricos apresentaram-se associados a outros marcadores de perfil lipídico, glicídico e de adiposidade, evidenciando a relevância da vigilância em saúde para o planejamento de ações efetivas para a reversão do quadro e prevenção de novos casos.

## Introdução

As doenças crônicas não transmissíveis (DCNTs) tornaram-se um problema de grande relevância para a saúde pública, protagonizando o cenário epidemiológico mundial junto às doenças cardiovasculares de ocorrência aguda.^1^ Entre as DCNTs mais prevalentes no mundo, destaca-se a hipertensão arterial sistêmica (HAS), uma condição clínica caracterizada por níveis elevados e sustentados da pressão arterial (PA), reconhecida por ser um importante fator de risco para doenças cardiovasculares, além de frequentemente estar associada a outros distúrbios metabólicos como obesidade, dislipidemias e intolerância à glicose.^2^

A Organização Mundial de Saúde (OMS) estimou, em 2010, que cerca de 600 milhões de pessoas tinham diagnóstico de HAS, predizendo um crescimento global de 60% dos casos até 2025.^3^ No Brasil, dados da Pesquisa Nacional de Saúde (PNS), realizada em 2013, mostraram uma prevalência de 21,4% da doença na população adulta.^4^ Paralelamente, tem sido observada uma mudança no perfil demográfico dos indivíduos com doenças crônicas, sendo cada vez mais comum a sua presença na infância e adolescência.^5^

As primeiras fases do curso da vida são importantes para o desenvolvimento humano, e alterações metabólicas precoces podem repercutir de maneira negativa na vida adulta, aumentando o risco para o desenvolvimento de doenças e comorbidades ao longo dos anos.^6^ O Estudo de Riscos Cardiovasculares em Adolescentes (ERICA), realizado com escolares de todas as regiões brasileiras nos anos de 2013 e 2014, estimou a prevalência da doença em 9,6% dos participantes.^7^ Uma revisão sistemática com metanálise, publicada em 2016,^5^ estimou a prevalência de HAS em 8% dos adolescentes brasileiros.

Tendo em vista esse cenário, a importância de monitorar o estado de saúde da população adolescente para auxiliar a tomada de decisão em ações de saúde e, ainda, a inexistência de estudos representativos sobre HAS e outros parâmetros metabólicos associados, conduzidos com a população adolescente do Distrito Federal (DF), o presente estudo se propõe a estimar a prevalência de HAS e investigar sua associação com parâmetros lipídicos, glicídicos e de adiposidade em adolescentes escolares do DF.

## Método

### Delineamento e contexto do estudo

Trata-se de um estudo observacional do tipo transversal realizado com participantes do ERICA entre 2013 e 2014.^8^

### Critérios de elegibilidade

Foram considerados elegíveis adolescentes de 12 a 17 anos, dos três últimos anos do Ensino Fundamental e do Ensino Médio de escolas públicas e privadas, localizadas em áreas rurais e urbanas, sem qualquer deficiência, provisória ou definitiva, que nunca engravidaram e que aceitaram participar das coletas de sangue.

### Tamanho e seleção da amostra

O ERICA teve representatividade em municípios de médio e grande porte, em nível nacional, regional e para cada capital incluída. Outros detalhes sobre a amostra nacional e a representatividade do estudo podem ser encontrados em Vasconcellos et al.^9^

No DF, a coleta de exames laboratoriais foi realizada em 33 escolas. Para a verificação da adequação do tamanho amostral para este estudo, considerou-se o total de 233.399 alunos no DF em 2009 nos últimos três anos do Ensino Fundamental e nos três anos do Ensino Médio,^10^ a prevalência de HAS na população adolescente escolar brasileira de 9%,^7^ erro aceitável de 1,7% e nível de confiança de 95%, totalizando número mínimo de 1.084 adolescentes.

### Variáveis

#### Pressão arterial

As medidas de pressão arterial sistólica (PAS) e diastólica (PAD) foram consideradas variáveis de desfecho. Foi utilizado o aparelho oscilométrico automático, validado para adolescentes, Omron^®^ 705-IT.^11^

Foram aferidas três medidas, com intervalo de 3 minutos entre cada uma; porém, foram consideradas apenas a média da segunda e da terceira medidas.^8^ Os adolescentes foram classificados de acordo com os valores de PAS e PAD em relação a estatura, sexo e idade, sendo ≥ percentil 95 classificados com HAS.^12^

#### Coleta de exames laboratoriais

A determinação dos parâmetros bioquímicos foi realizada a partir da coleta de amostra sanguínea por punção venosa, com jejum de 12 horas.^13^ A glicose sanguínea foi determinada a partir do método hexoquinase, e considerou-se valores ≥ 100 mg/dL elevados.^14^ A hemoglobina glicada (HbA1c) foi estimada por cromatografia de troca iônica, sendo consideradas elevadas as concentrações ≥ 5,8%, valor correspondente ao percentil 90 para a população estudada. A insulina foi determinada pelo método de quimiluminescência e considerada elevada quando ≥ 15 mU/L.^15^

O modelo de avaliação da homeostase da resistência à insulina (HOMA-IR) foi utilizado para caracterizar a resistência à insulina (RI)^16^ e calculado com da seguinte fórmula: insulina de jejum (mU/L) x (glicose de jejum [mg/dL] x 0,0555)/22,5. Foram considerados elevados os valores de HOMA-IR ≥ 2,80.^17^

O colesterol total (CT) e os triglicerídeos (TG) foram determinados pelo método de cinética enzimática, sendo consideradas elevadas as dosagens de CT ≥ 170 mg/dL e TG ≥ 90 mg/dL.^18^ Avaliou-se, ainda, a lipoproteína de baixa densidade (LDL) e lipoproteína de alta densidade (HDL), sendo empregado o ensaio calorimétrico enzimático. Para LDL, os valores considerados alterados foram ≥ 110 mg/dL; para HDL, ≤ 45 mg/dL.^18^

#### Parâmetros relacionados à adiposidade corporal

Para aferição de peso e estatura, foram utilizados, respectivamente, balança eletrônica (Líder^®^), com capacidade de 200 kg e precisão de 50g, e estadiômetro portátil (Alturexata^®^), com precisão de 1 mm e campo de uso de até 213 cm. A estatura foi aferida em duplicata, permitindo a variação máxima de 0,5 cm entre as duas medidas. A média foi calculada automaticamente por sistema desenvolvido para uso em *personal digital assistant* (PDA).^8^

O índice de massa corporal (IMC) foi calculado a partir da divisão do peso (em quilogramas) pelo quadrado da altura (em metros). Foram utilizadas as referências da OMS^19^ para o cálculo dos escores-Z de IMC/idade, considerando o sexo. Foram considerados os seguintes pontos de corte: escore-Z < −2 (baixo peso); escore-Z ≥ −2 e < 1 (eutrofia); escore-Z ≥ 1 e < 2 (sobrepeso); escore-Z ≥ 2 (obesidade).

Para aferição do perímetro da cintura (PC), foi utilizada fita antropométrica com resolução milimétrica e 1,5 m de comprimento (Sanny^®^), adotando-se a técnica do ponto médio entre a crista ilíaca e a menor margem costal. A medida foi coletada em duplicata, e a média foi calculada.^8^ Os pontos de corte considerados foram os valores ≥ percentil 90 para a população estudada.

#### Variáveis demográficas e econômicas

As variáveis foram autorreferidas e assim categorizadas: sexo (feminino e masculino), idade (< 15 e ≥ 15 anos) e cor da pele ou etnia (brancos, pardos, negros, indígenas, asiáticos e não referido). A localização (rural e urbana) e a rede de ensino (pública e privada) da escola foram identificadas, sendo essa última variável utilizada como *proxy* da classe econômica da família.

#### Maturação sexual

Os adolescentes foram classificados em diferentes estágios de maturação sexual, de acordo com o instrumento proposto por Tanner.^20^ Categorizou-se a partir da característica mais desenvolvida apontada, sendo os estágios 4 e 5 classificados como púberes e os demais, como não púberes.

## Análise dos dados

Na etapa descritiva, calculou-se a prevalência e a distribuição das características de interesse na população estudada, bem como a prevalência de HAS em relação a essas características. Também foi realizada a comparação da prevalência de alterações em parâmetros bioquímicos e antropométricos entre os adolescentes com e sem HAS. Os resultados foram apresentados junto aos seus intervalos de confiança de 95% (IC 95%).

Na fase analítica, utilizou-se a técnica de regressão linear para investigar a associação entre PAS e PAD (variáveis dependentes) e marcadores laboratoriais e antropométricos (variáveis independentes). Essa fase foi dividida em duas etapas, análise bruta e análise ajustada, e foram utilizadas as seguintes variáveis para o ajuste: sexo, idade, estágio de maturação sexual, cor da pele ou etnia, obesidade e rede da escola. Quando a variável independente se referiu ao IMC ou ao PC, não houve ajuste pelo *status* de obesidade. Os resultados foram apresentados por meio do coeficiente β da regressão com IC 95%. A análise ajustada foi realizada somente quando a análise bruta apresentou p < 0,20, e considerou-se significativo p < 0,05.

O desenho da amostra complexa e os respectivos pesos amostrais relacionados à população adolescente estudante do DF foram considerados. Foi utilizado o *software* Stata, versão 14.2.

## Aspectos éticos

O projeto foi aprovado pelo Comitê de Ética em Pesquisa com Seres Humanos da Faculdade de Medicina, Universidade de Brasília (CAAE N° 05185212.2.2005.5540). Os participantes foram previamente informados acerca dos objetivos e procedimentos da pesquisa e foram avaliados somente após assinados os Termos de Assentimento pelo estudante e de Consentimento Livre e Esclarecido por seus pais ou responsáveis.

## Resultados

Foram avaliados 1.200 adolescentes de 33 escolas públicas e privadas do Distrito Federal (DF). A média de idade foi de 14,8 anos, e a prevalência de HAS, de 8,0% (IC 95%: 6,3; 9,9), sendo identificada com maior frequência em estudantes do sexo masculino, com idade ≥ 15 anos e que estudavam em escolas da zona rural.

A análise dos marcadores sanguíneos revelou a hiperglicemia como a alteração de menor prevalência. A alteração mais prevalente foi o baixo valor de HDL. Outras características estão descritas na Tabela 1 .

**Tabela 1:** – Perfil dos adolescentes escolares e prevalência de hipertensão arterial sistêmica. Estudo de Riscos Cardiovasculares em Adolescentes, Distrito Federal, Brasil, 2013–2014

	Amostra total		Adolescentes com HAS	
Característica	%	IC 95%	%	IC 95%
Localização da escola				
Área urbana	97,0	81,4; 99,5	7,4	5,9; 9,0
Área rural	3,0	0,4; 18,5	27,7	24,6; 30,8
**Rede de ensino**				
Rede pública	55,2	37,5; 71,5	8,3	5,8; 11,7
Rede privada	44,8	28,4; 62,4	7,6	5,7; 9,8
**Sexo**				
Feminino	50,4	-	4,3	2,8; 6,2
Masculino	49,6	-	11,8	9,3; 14,7
**Idade**				
< 15 anos	47,6	-	5,4	3,8; 7,6
≥ 15 anos	52,4	-	10,3	7,7; 13,5
**Cor da pele/etnia**				
Brancos	35,6	30,1; 41,3	8,4	5,3; 13,1
Pardos	53,5	48,4; 58,4	7,9	6,1; 10,1
Negros	6,0	4,4; 8,2	8,9	3,9; 19,1
Indígenas	0,2	0,0; 0,6	16,7	1,5; 71,5
Asiáticos	2,7	1,7; 4,1	3,5	0,8; 13,6
Não referido	2,0	1,3; 3,1	2,8	0,3; 18,7
Estágio de maturação sexual*				
Púbere	81,9	78,2; 85,0	8,0	6,3; 10,1
Pré-púbere	18,1	14,9; 21,7	7,7	3,6; 15,5
**Glicose sanguínea^†^**				
≥ 100 mg/dL	1,5	0,7; 3,2	28,1	8,4; 62,4
**HbA1c^‡^**				
≥ 5,8% (≥ p90)	13,6	10,7; 17,1	7,5	3,6; 14,8
**Insulina^§^**				
≥ 15 mU/L	11,3	8,2; 15,5	17,6	11,6; 25,8
**HOMA-IR^//^**				
≥ 2,80	18,2	13,9; 23,5	15,7	10,7; 22,4
**Triglicerídeos^¶^**				
≥ 90 mg/dL	30,5	27,4; 33,8	9,4	6,3; 14,0
**Colesterol total^¶^**				
≥ 170 mg/dL	30,6	27,6; 33,7	9,2	5,9; 13,8
**LDL^¶^**				
≥110 mg/dL	21,3	19,0; 23,7	7,2	4,7; 10,8
**HDL^¶^**				
≤ 45 mg/dL	41,8	38,1; 45,4	9,5	7,2; 12,5
**IMC^#^**				
Baixo peso e eutrofia	77,0	73,8; 79,9	4,2	2,9; 6,0
Sobrepeso	14,7	12,3; 17,4	16,0	10,8; 23,0
Obesidade	8,3	6,3; 10,6	28,6	18,2; 41,9
Perímetro da cintura^**^				
Não elevado (< p90)	88,4	84,8; 91,1	6,2	4,8; 7,8
Elevado (≥ p90)	11,6	8,8; 15,1	21,6	14,2; 31,3

HAS: hipertensão arterial sistêmica; IC 95%: intervalo de confiança de 95%; HbA1c: hemoglobina glicada; HOMA-IR: modelo de avaliação da homeostase da resistência à insulina; LDL: lipoproteína de baixa densidade; HDL: lipoproteína de alta densidade; IMC: índice de massa corporal. *Tanner, 1962; ^**†**^ SBD, 2019; ^**‡**^ valores ≥ 5,8% (correspondente ao percentil 90 para a população estudada); ^**§**^ SBC, 2005; ^**//**^ Chissini et al., 2019; ^**¶**^ SBC, 2017; ^**#**^ OMS, 2007; **valores ≥ 80,8 cm (feminino) ou ≥ 86,3 cm (masculino).

Observou-se maior prevalência de hiperinsulinemia em adolescentes com HAS. Os parâmetros de adiposidade foram maiores em escolares com HAS, quando comparados aos sem HAS ( Tabela 2 ).

**Tabela 2:** – Prevalência de alterações bioquímicas e do estado nutricional em adolescentes com e sem hipertensão arterial sistêmica. Estudo de Riscos Cardiovasculares em Adolescentes, Distrito Federal, Brasil, 2013–2014

	Adolescentes com HAS		Adolescentes sem HAS	
Parâmetro avaliado	%	IC 95%	%	IC 95%
Glicose sanguínea*				
≥ 100 mg/dL	5,4	1,3; 18,8	1,1	0,5; 2,5
**HbA1c^†^**				
≥ 5,8% (≥ p90)	12,7	6,0; 25,1	13,7	10,9; 17,1
**Insulina^‡^**				
≥ 15 mU/L	25,4	13,8; 41,8	10,2	7,5; 13,6
**HOMA-IR^§^**				
≥ 2,80	35,9	23,8; 50,1	16,7	12,7; 21,6
**Triglicerídeos^//^**				
≥ 90 mg/dL	36,3	26,3; 47,6	30,0	27,0; 33,2
**Colesterol total^//^**				
≥ 170 mg/dL	35,1	23,8; 48,4	30,2	27,1; 33,4
**LDL^//^**				
≥110 mg/dL	19,2	12,2; 28,9	21,4	19,2; 23,9
**HDL^//^**				
≤ 45 mg/dL	50,0	36,3; 63,8	41,0	37,6; 44,5
**IMC^¶^**				
Baixo peso e eutrofia	40,7	29,1; 53,4	80,1	77,0; 82,9
Sobrepeso	29,6	20,3; 41,0	13,4	11,0; 16,1
Obesidade	29,6	18,5; 43,8	6,4	4,7; 8,5
**Perímetro da cintura^#^**				
Elevado (≥ p90)	31,5	20,0; 45,8	9,9	7,5; 12,8

HAS: hipertensão arterial sistêmica; IC 95%: intervalo de confiança de 95%; HbA1c: hemoglobina glicada; HOMA-IR: modelo de avaliação da homeostase da resistência à insulina; LDL: lipoproteína de baixa densidade; HDL: lipoproteína de alta densidade; IMC: índice de massa corporal. ^*^SBD, 2019; ^†^valores ≥ 5,8% (correspondente ao percentil 90 para a população estudada); ^‡^SBC, 2005; ^**§**^ Chissini et al., 2019;^**//**^ SBC, 2017; ^**¶**^ OMS, 2007; ^#^Valores ≥ 80,8 cm para sexo feminino ou ≥ 86,3 cm para sexo masculino (correspondente ao percentil 90 para a população estudada).

A maior parte dos parâmetros analisados se associou à PAS e PAD na análise bruta. Na análise ajustada, parâmetros glicídicos, lipídicos e de adiposidade mantiveram-se associados, tendo o IMC e o HOMA-IR apresentado maior magnitude nessa relação ( Tabela 3 ).

**Tabela 3:** – Associação entre parâmetros bioquímicos e de estado nutricional e a pressão arterial sistólica e diastólica em adolescentes. Estudo de Riscos Cardiovasculares em Adolescentes, Distrito Federal, Brasil, 2013–2014

Parâmetro avaliado	Análise bruta		Análise ajustada*	
	Coeficiente β	IC 95%	Coeficiente β	IC 95%
**PAS**				
Glicose (mg/dL)	0,26^§^	0,13; 0,39	0,16^‡^	0,04; 0,27
HbA1c (%)	1,77^†^	-0,84; 4,39	0,68	-1,41; 2,78
Insulina (mU/L)	0,24^‡^	0,03; 0,45	0,23^‡^	0,06; 0,40
HOMA-IR	1,19^‡^	0,17; 2,21	1,07^‡^	0,25; 1,88
TG (mg/dL)	0,04^§^	0,02; 0,06	0,03^‡^	0,01; 0,04
CT (mg/dL)	-0,001	-0,03; 0,02		
LDL (mg/dL)	0,008	-0,02; 0,03		
HDL (mg/dL)	-0,18^§^	-0,27; -0,09	-0,06^‡^	-0,12; -0,002
IMC (kg/m^2^)	1,49^§^	1,28; 1,71	1,41^§^	1,20; 1,63
PC (cm)	0,68^§^	0,58; 0,79	0,58^§^	0,48; 0,69
**PAD**				
Glicose (mg/dL)	0,10^‡^	0,01; 0,19	0,08^‡^	0,002; 0,17
HbA1c (%)	1,09^†^	-0,56; 2,76	0,65	-0,77; 2,07
Insulina (mU/L)	0,13^†^	-0,05; 0,31	0,09	-0,05; 0,24
HOMA-IR	0,62^†^	-0,26; 1,51	0,45	-0,25; 1,16
TG (mg/dL)	0,02^‡^	0,01; 0,03	0,01^‡^	0,005; 0,02
CT (mg/dL)	0,02^‡^	0,00; 0,03	0,02^‡^	0,007; 0,03
LDL (mg/dL)	0,01^‡^	0,00; 0,03	0,01^‡^	0,002; 0,03
HDL (mg/dL)	-0,01	-0,06; 0,02		
IMC (kg/m^2^)	0,57^§^	0,43; 0,72	0,56^§^	0,40; 0,72
PC (cm)	0,27^§^	0,20; 0,33	0,25^§^	0,18; 0,33

IC 95%: intervalo de confiança de 95%; PAS: pressão arterial sistólica; HbA1c: hemoglobina glicada; HOMA-IR: modelo de avaliação da homeostase da resistência à insulina; TG: triglicerídeos; CT: colesterol total; LDL: lipoproteína de baixa densidade; HDL: lipoproteína de alta densidade; IMC: índice de massa corporal; PC: perímetro da cintura; PAD: pressão arterial diastólica. ^†^ p < 0,20; ^‡^ p < 0,05; ^§^ p < 0,001; * A análise foi ajustada para sexo, idade, estágio de maturação sexual, cor da pele ou etnia, rede de ensino e presença ou não de obesidade.

## Discussão

Este é o primeiro estudo a investigar HAS nos adolescentes escolares do DF, e a prevalência estimada foi semelhante à encontrada para as regiões Centro-oeste (8,7, IC 95%: 7,9; 9,6), Norte (8,4, IC 95%: 7,7; 9,2), Nordeste (8,4, IC 95%: 7,6; 9,2), Sudeste (9,8, IC 95%: 8,8; 11,0) e para a amostra nacional do ERICA (9,6, IC 95%: 9,0; 10,3). Foi menor somente que a prevalência estimada para a região Sul (12,5, IC 95%: 11,0; 14,2).^7^ Foi verificada, ainda, elevada prevalência de alterações nos demais parâmetros bioquímicos e de adiposidade investigados, e as associações encontradas podem potencializar o risco cardiovascular nessa população.

De maneira semelhante à amostra nacional do ERICA,^7^ apesar de a maioria dos adolescentes estudarem na zona urbana, a HAS estava mais presente em escolas da zona rural. Uma hipótese explicativa relaciona-se ao fato de os ambientes rurais muitas vezes terem acesso limitado a serviços de saúde, dificultando o diagnóstico e tratamento de doenças crônicas como a HAS.^21^

A avaliação de parâmetros relacionados ao metabolismo glicídico dos adolescentes mostrou que alterações na glicemia em jejum foram menos prevalentes que nos demais parâmetros. No entanto, a avaliação isolada da glicemia é insuficiente para descartar alterações metabólicas, visto que, no início do quadro de RI, pode ocorrer a manutenção da glicemia dentro dos níveis de normalidade como consequência de uma possível hiperinsulinemia.^14^ A alteração na HbA1c, que foi mais prevalente nesses estudantes, pode ser um melhor marcador na avaliação do controle glicêmico, por refletir mudanças na glicemia a longo prazo.^22^

Níveis elevados de glicose sanguínea favorecem a HAS por meio do aumento de débito cardíaco causado pela hiperosmolaridade induzida pelo quadro de hiperglicemia.^23^ O excesso de glicose sanguínea também pode acarretar a geração excessiva de espécies reativas de oxigênio (EROs), o que colabora com a disfunção endotelial.^24^ Quando prolongada, a hiperglicemia pode contribuir, ainda, para a geração de produtos finais de glicação avançada, que pioram o estresse oxidativo por ativar uma cascata pró-inflamatória, aumentando a expressão de EROs e contribuindo para inibição ou redução da produção de óxido nítrico, o que leva a resistência vascular periférica por vasoconstrição.^24 , 25^

Além disso, verificou-se elevação nos níveis de insulina e HOMA-IR. Andrade et al.^26^ sugerem, no entanto, que a RI pode estar relacionada ao desenvolvimento na adolescência, envolvendo alterações hormonais e de composição corporal em indivíduos de estágios iniciais da puberdade, podendo ser revertida após o estirão de crescimento. No entanto, isso não explica os resultados encontrados, pois a maior parte da população estudada encontrava-se na fase final da puberdade. Assim como entre os adolescentes do DF, outros estudos identificaram maiores níveis de insulina e alterações no HOMA-IR em adolescentes com HAS em relação aos demais.^27 , 28^

Os adolescentes com HAS também apresentaram maior IMC e PC, situação bem estabelecida em outros estudos.^29 , 30^ A adiposidade elevada contribui para o quadro de hipertensão, entre outros mecanismos, por favorecer o estresse oxidativo a partir da instalação de um estado pró-inflamatório com aumento na expressão de citocinas como interleucina 6 (IL-6) e fator de necrose tumoral α.^31^ A inflamação é um mediador importante tanto na instalação quanto na manutenção dos níveis pressóricos elevados pela lesão vascular e renal que pode causar.^32^ Além disso, indivíduos com obesidade podem apresentar hiperativação simpática, com aumento na produção de noradrenalina em nível renal e consequente aumento na reabsorção tubular de sódio.^33^ A hiperativação simpática pode ser ainda mais estimulada pela produção excessiva de leptina, comum em indivíduos com excesso de adiposidade.^34^ A presença de tecido adiposo não funcionante observada na obesidade pode alterar, ainda, o sistema renina angiotensina, aumentando os níveis circulantes de angiotensina II e aldosterona, causando alterações hemodinâmicas que também contribuem para a elevação da PA,^35^ justificando a associação observada no estudo.

A presença de marcadores de inflamação e disfunção endotelial também são perfis característicos das dislipidemias.^36^ Alterações de perfil lipídico e presença de citocinas inflamatórias como IL-6 se relacionam com o aumento da rigidez arterial e consequentemente com a pressão sanguínea, favorecendo a instalação do quadro hipertensivo e aumentando ainda o risco do desenvolvimento de doenças cardiovasculares.^37^

Estudos realizados em outras regiões do Brasil^29 , 38^ e do mundo^39 , 40^ corroboram as prevalências e associações encontradas no DF, reforçando a frequência de fatores associados ao risco cardiovascular em idades cada vez mais precoces.^41^ O estilo de vida não saudável da população adolescente brasileira, principalmente representado pela baixa qualidade da alimentação,^42 , 43^ sedentarismo e elevado tempo de tela,^44^ além do estresse emocional,^45^ potencializa os riscos apresentados.

A interpretação dos resultados do ERICA, no entanto, deve levar em consideração algumas de suas limitações. A prevalência de HAS pode ter sido superestimada pelo fato de a pressão ter sido aferida em um único dia.^12^ Em estudos transversais de grande magnitude como o ERICA essa limitação é frequente, já que aumentar o número de visitas implica em maiores investimentos financeiros e logísticos. Vale ressaltar que a própria metodologia adotada no dia de coleta pode ter reduzido esse viés.^8^

Apesar dessa limitação, o ERICA contou com processos de monitoramento de qualidade ao longo de todo o período de coleta, além de grande cuidado metodológico adotado durante a análise estatística. Isso contribuiu para a robustez do estudo e a confiabilidade dos resultados encontrados.

## Conclusão

A prevalência de HAS estimada em adolescentes escolares do DF foi de 8%, associada a parâmetros metabólicos e de adiposidade. Esses achados evidenciam as interconexões metabólicas que podem estar presentes no quadro clínico hipertensivo. Nesse contexto, ações de promoção à saúde e prevenção de doenças são fundamentais para se evitar a situação evidenciada no DF, colaborando com a qualidade de vida da população e com menor impacto ao sistema de saúde vigente.
