# Supplementary material for: Hipertensão Arterial e Parâmetros Lipídicos, Glicídicos e de Adiposidade Associados em Adolescentes Escolares do Distrito Federal
Source: Arq Bras Cardiol. 2022 Jan 11;118(4):719–26. [Article in Portuguese] doi: 10.36660/abc.20201240 (PMC9007003; doi:10.36660/abc.20201240)
Supplement: Translation

# Translation: Hypertension and Associated Lipid, Glucose, and Adiposity Parameters in School-Aged Adolescents in the Federal District, Brazil

**Authors:** Letícia Rocha Lima, Aline Bassetto Okamura, Kênia Mara Baiocchi de Carvalho, Eliane Said Dutra, Vivian Siqueira Santos Gonçalves

**Affiliations:** 1 Brasil Departamento de Nutrição - Universidade de Brasília, Brasília, DF – Brasil; 2 Brasil Programa de Pós-graduação em Saúde Coletiva - Universidade de Brasília, Brasília, DF – Brasil; 3 Brasil Programa de Pós-graduação em Nutrição Humana - Universidade de Brasília, Brasília, DF – Brasil

**Keywords:** Hypertension, Adolescent, Adiposity, Blood Glucose, Lipids

## Abstract

**Background:**

The prevalence of hypertension and other metabolic disorders has increased in young individuals. However, no representative studies have been conducted in the population of the Federal District, Brazil.

**Objective:**

To estimate the prevalence of hypertension and its association with lipid, glucose, and adiposity markers in school-aged adolescents living in the Federal District.

**Methods:**

This cross-sectional study included participants of the Study of Cardiovascular Risks in Adolescents (Portuguese acronym, ERICA). Blood pressure, blood glucose, glycated hemoglobin, insulin, homeostatic model assessment for insulin resistance (HOMA-IR), triglycerides, total cholesterol, high-density lipoprotein, low-density lipoprotein, body mass index (BMI), waist circumference, and economic, demographic, and sexual maturity variables were assessed. The data were analyzed in Stata, and the analysis was divided into different stages: descriptive, crude, and adjusted. Significant results were set at p < 0.05.

**Results:**

In total, 1,200 adolescents were included, and their mean age was 14.8 years. The prevalence of hypertension was 8% (95% confidence interval: 6.3; 9.9). Most parameters were associated with blood pressure in crude analysis. In adjusted analysis, glucose, lipid, and adiposity markers maintained the associations, and the highest magnitudes were those of BMI and HOMA-IR.

**Conclusion:**

The study revealed a high prevalence of hypertension in adolescents living in the Federal District, and blood pressure levels were associated with other markers of lipid, glucose, and adiposity profile. The findings indicate the relevance of health surveillance for planning effective actions aimed at reversing this situation and preventing new cases.

## Introduction

Noncommunicable diseases (NCDs) have become a public health problem of great relevance, playing a leading role in the global epidemiological setting together with acute cardiovascular diseases.^1^ One of the most prevalent NCDs in the world is hypertension, a clinical condition characterized by high and sustained levels of blood pressure. It is known as an important risk factor for cardiovascular disease, in addition to being frequently associated with other metabolic disorders such as obesity, dyslipidemia, and glucose intolerance.^2^

The World Health Organization (WHO) reported in 2010 that an estimated 600 million people had a diagnosis of hypertension, predicting a 60% global increase in the number of cases by 2025.^3^ In Brazil, 2013 National Health Survey data showed a prevalence of 21.4% for hypertension in the adult population.^4^ A concomitant change in the demographic profile of individuals with chronic diseases has been observed, and their presence in children and adolescents is increasingly common.^5^

The first stages of life are important for human development, and early metabolic changes can have a negative impact on adulthood, increasing the risk of developing diseases and comorbidities over the years.^6^ The Study of Cardiovascular Risks in Adolescents (Portuguese acronym, ERICA), which evaluated students from all Brazilian regions during 2013 and 2014, reported an estimated prevalence of 9.6% for hypertension.^7^ A 2016 systematic review with meta-analysis^5^ described an estimated prevalence of 8% for hypertension in Brazilian adolescents.

Given the importance of monitoring the health status of the adolescent population to assist health care decision-making and the lack of representative studies on hypertension and associated metabolic parameters in the adolescent population of the Federal District, Brazil, this study aimed to estimate the prevalence of hypertension and investigate its association with lipid, glucose, and adiposity parameters in Federal District school-aged adolescents.

## Methods

### Study design and setting

This cross-sectional study included participants of the ERICA study, conducted during 2013 and 2014.^8^

### Eligibility criteria

Adolescents aged 12 to 17 years attending the final three years of middle school and high school in public and private institutions located in rural and urban areas, without any temporary or permanent disability, who had never become pregnant, and who agreed to participate in blood specimen collections were defined as eligible.

### Sample size and participant selection

ERICA was representative of the adolescent population in large and medium-sized municipalities at the national, regional, and capital levels. Further details about the national sample and the representativeness of the study can be found in Vasconcellos et al.^9^

In the Federal District, blood samples were collected for laboratory testing at 33 schools. The adequacy of sample size for this study was ascertained by calculations including a total of 233,399 students in the Federal District attending in 2009 the final three years of middle school and the three years of high school,^10^ a prevalence of 9% for hypertension in the Brazilian school-aged adolescent population,^7^ an acceptable error of 1.7%, and a 95% confidence level. Thus, the minimum number of adolescents was 1,084.

### Variables

#### Blood pressure

Systolic and diastolic blood pressure (SBP and DBP, respectively) measurements were defined as outcome variables. Omron® 705-IT, an automatic oscillometric device validated for adolescents, was used.^11^

Three measurements were taken, with a 3-minute interval between each one, but only the mean of the second and third measurements was used.^8^ Adolescents were classified according to SBP and DBP values in relation to height, sex, and age, and those with values ≥ 95th percentile were defined as having hypertension.^12^

#### Bood samples collection for laboratory testing

Blood samples were collected by venipuncture after a 12-hour fasting period for determination of biochemical markers.^13^ Blood glucose was determined by the hexokinase method, and values ≥ 100 mg/dL were defined as high.^14^ Glycated hemoglobin (HbA1c) was measured by ion exchange chromatography, and concentrations ≥ 5.8%, corresponding to the 90th percentile for the study population, were defined as high. Insulin was determined by the chemiluminescence method and defined as high if ≥ 15 mU/L.^15^

Homeostatic model assessment for insulin resistance (HOMA-IR) was used to characterize insulin resistance (IR)^16^ and calculated as follows: fasting insulin (mU/L) x (fasting glucose (mg/dL) x 0.0555)/22.5. HOMA-IR values ≥ 2.80 were defined as high.^17^

Total cholesterol (TC) and triglycerides (TG) were determined by an enzyme kinetic assay, and TC ≥ 170 mg/dL and TG ≥ 90 mg/dL were defined as high.^18^ Low-density lipoprotein (LDL) and high-density lipoprotein (HDL) were assessed with an enzymatic colorimetric assay, and LDL ≥ 110 mg/dL and HDL ≤ 45 mg/dL were defined as abnormal.^18^

#### Adiposity markers

An electronic scale (Líder®) with a capacity of 200 kg and a precision of 50 g was used to measure weight, and a portable stadiometer (Alturexata®) with a precision of 1 mm and a range of up to 213 cm was used to measure height. Height was measured twice, and the maximum variation between the two measurements should be 0.5 cm. Mean was calculated automatically by a system developed for use in a personal digital assistant (PDA).^8^

Body mass index (BMI) was calculated by dividing weight (kg) by height squared (m). WHO references^19^ were used to calculate BMI-for-age z-scores adjusted for sex. The following cutoff points were used: z-score < -2, underweight; z-score ≥ -2 and < 1, normal weight; z-score ≥ 1 and < 2, overweight; and z-score ≥ 2, obesity.

A measuring tape with a resolution in millimeters and 1.5 m in length (Sanny®) was used to measure waist circumference (WC) at the midpoint between the iliac crest and the lowest rib. Measures were collected twice, and means were calculated.^8^ Cutoff points were taken as values ≥ 90th percentile for the study population.

#### Demographic and economic variables

The variables were self-reported and categorized as follows: sex (female or male), age (< 15 or ≥ 15 years), and skin color or ethnicity (White, Brown, Black, Indigenous, Asian, or not reported). The schools were classified according to setting (rural or urban) and type (public or private), and the latter was used as a proxy for the family’s economic class.

#### Sexual maturity

Adolescents were rated at different stages of sexual maturity according to the Tanner staging scale.^20^ The most developed characteristic was used for categorization, and stages 4 and 5 were defined as pubescent and the others as prepubescent.

## Data analysis

In the descriptive stage, the prevalence and distribution of characteristics of interest in the study population were calculated, as well as the prevalence of hypertension in relation to these characteristics. Also, the prevalence of differences in biochemical and anthropometric parameters was compared between adolescents with and without hypertension. The results were accompanied by their respective 95% confidence intervals (CIs).

In the analytical stage, linear regression was used to investigate the associations between SBP and DBP (dependent variables) and laboratory and anthropometric markers (independent variables). This stage was subdivided into crude analysis and adjusted analysis, and the following variables were used for adjustment: sex, age, sexual maturity stage, skin color or ethnicity, obesity, and type of school. When the independent variable referred to BMI or WC, no adjustment was made for obesity status. The results were reported as β coefficients with their respective 95% CIs. Adjusted analysis was performed only when crude analysis presented p < 0.20, and p < 0.05 was defined as significant.

The complex sample design and the respective sample weights referring to the Federal District school-aged adolescent population were accounted for. Stata version 14.2 was used for all analyses.

## Ethical aspects

The project was approved by the Human Research Ethics Committee of Faculty of Medicine, Universidade de Brasília (certificate number 05185212.2.2005.5540). The participants were previously informed of the study objectives and procedures and were evaluated only after assent and consent forms were signed by the students and by their parents or guardians, respectively.

## Results

In total, 1,200 adolescents studying at 33 public and private schools in the Federal District were evaluated. The mean age was 14.8 years, and the prevalence of hypertension was 8.0% (95% CI: 6.3; 9.9). Hypertension was more frequently found in male students, in those aged ≥ 15 years, and in those studying at rural schools.

The analysis of blood markers revealed that hyperglycemia was the least prevalent inadequacy. The most prevalent inadequacy was low HDL value. Other characteristics are described in Table 1 .

**Table 1:** – Profile of school-aged adolescents and prevalence of hypertension. Study of Cardiovascular Risks in Adolescents, Federal District, Brazil, 2013-2014

	Total sample		With hypertension	
Characteristic	%	95% CI	%	95% CI
School setting				
Urban area	97.0	81.4; 99.5	7.4	5.9; 9.0
Rural area	3.0	0.4; 18.5	27.7	24.6; 30.8
**Type of school**				
Public	55.2	37.5; 71.5	8.3	5.8; 11.7
Private	44.8	28.4; 62.4	7.6	5.7; 9.8
**Sex**				
Female	50.4	-	4.3	2.8; 6.2
Male	49.6	-	11.8	9.3; 14.7
**Age**				
< 15 years	47.6	-	5.4	3.8; 7.6
≥ 15 years	52.4	-	10.3	7.7; 13.5
**Skin color/ethnicity**				
White	35.6	30.1; 41.3	8.4	5.3; 13.1
Brown	53.5	48.4; 58.4	7.9	6.1; 10.1
Black	6.0	4.4; 8.2	8.9	3.9; 19.1
Indigenous	0.2	0.0; 0.6	16.7	1.5; 71.5
Asian	2.7	1.7; 4.1	3.5	0.8; 13.6
Not reported	2.0	1.3; 3.1	2.8	0.3; 18.7
Sexual maturity stage*				
Pubescent	81.9	78.2; 85.0	8.0	6.3; 10.1
Prepubescent	18.1	14.9; 21.7	7.7	3.6; 15.5
**Blood glucose^†^**				
≥ 100 mg/dL	1.5	0.7; 3.2	28.1	8.4; 62.4
**HbA1c^‡^**				
≥ 5.8% (≥ 90th pctl)	13.6	10.7; 17.1	7.5	3.6; 14.8
**Insulin^§^**				
≥ 15 mU/L	11.3	8.2; 15.5	17.6	11.6; 25.8
**HOMA-IR^//^**				
≥ 2.80	18.2	13.9; 23.5	15.7	10.7; 22.4
**Triglycerides^¶^**				
≥ 90 mg/dL	30.5	27.4; 33.8	9.4	6.3; 14.0
**Total cholesterol^¶^**				
≥ 170 mg/dL	30.6	27.6; 33.7	9.2	5.9; 13.8
**LDL^¶^**				
≥ 110 mg/dL	21.3	19.0; 23.7	7.2	4.7; 10.8
**HDL^¶^**				
≤ 45 mg/dL	41.8	38.1; 45.4	9.5	7.2; 12.5
**BMI^#^**				
Underweight and normal weight	77.0	73.8; 79.9	4.2	2.9; 6.0
Overweight	14.7	12.3; 17.4	16.0	10.8; 23.0
Obesity	8.3	6.3; 10.6	28.6	18.2; 41.9
Waist circumference^**^				
Not increased (< 90th pctl)	88.4	84.8; 91.1	6.2	4.8; 7.8
Increased (≥ 90th pctl)	11.6	8.8; 15.1	21.6	14.2; 31.3

BMI: body mass index; CI: confidence interval; HbA1c: glycated hemoglobin; HDL: high-density lipoprotein; HOMA-IR: homeostatic model assessment for insulin resistance; LDL: low-density lipoprotein; pctl: percentile.^*^Tanner, 1962;^†^SBD, 2019;^‡^Values ≥ 5.8% (corresponding to the 90th percentile for the study population);^§^SBC, 2005;^//^Chissini et al., 2019;^¶^SBC, 2017;^#^WHO, 2007;^**^Values ≥ 80.8 cm (female) or ≥ 86.3 cm (male).

There was a higher prevalence of hyperinsulinemia in adolescents with hypertension. Adiposity parameters were higher in students with hypertension compared with those without hypertension ( Table 2 ).

**Table 2:** – Prevalence of biochemical and nutritional status changes in adolescents with and without hypertension. Study of Cardiovascular Risks in Adolescents, Federal District, Brazil, 2013-2014

	Adolescents with hypertension		Adolescents without hypertension	
Parameter	%	95% CI	%	95% CI
Blood glucose*				
≥ 100 mg/dL	5.4	1.3; 18.8	1.1	0.5; 2.5
**HbA1c^†^**				
≥ 5.8% (≥ 90th pctl)	12.7	6.0; 25.1	13.7	10.9; 17.1
**Insulin^‡^**				
≥ 15 mU/L	25.4	13.8; 41.8	10.2	7.5; 13.6
**HOMA-IR^§^**				
≥ 2.80	35.9	23.8; 50.1	16.7	12.7; 21.6
**Triglycerides^//^**				
≥ 90 mg/dL	36.3	26.3; 47.6	30.0	27.0; 33.2
**Total cholesterol^//^**				
≥ 170 mg/dL	35.1	23.8; 48.4	30.2	27.1; 33.4
**LDL^//^**				
≥ 110 mg/dL	19.2	12.2; 28.9	21.4	19.2; 23.9
**HDL^//^**				
≤ 45 mg/dL	50.0	36.3; 63.8	41.0	37.6; 44.5
**BMI^¶^**				
Underweight and normal weight	40.7	29.1; 53.4	80.1	77.0; 82.9
Overweight	29.6	20.3; 41.0	13.4	11.0; 16.1
Obesity	29.6	18.5; 43.8	6.4	4.7; 8.5
**Waist circumference^#^**				
Increased (≥ 90th pctl)	31.5	20.0; 45.8	9.9	7.5; 12.8

BMI: body mass index; CI: HbA1c: glycated hemoglobin; HDL: high-density lipoprotein; HOMA-IR: homeostatic model assessment for insulin resistance; LDL: low-density lipoprotein; pctl: percentile^*^SBD, 2019;^†^values ≥ 5,8% (corresponding to the 90th percentile for the study population);^‡^SBC, 2005;^§^Chissini et al., 2019;^//^SBC, 2017;^**¶**^ WHO, 2007;^#^Values ≥ 80,8 cm for female or ≥ 86,3 cm for male (corresponding to the 90th percentile for the study population).

Most parameters were associated with SBP and DBP in crude analysis. In adjusted analysis, glucose, lipid, and adiposity parameters maintained the associations, and the highest magnitudes were those of BMI and HOMA-IR ( Table 3 ).

**Table 3:** – Association between biochemical and nutritional status parameters and systolic and diastolic blood pressure in adolescents. Study of Cardiovascular Risks in Adolescents, Federal District, Brazil, 2013-2014

	Crude analysis		Adjusted analysis*	
Parameter	β coefficient	95% CI	β coefficient	95% CI
**SBP**				
Glucose (mg/dL)	0.26^§^	0.13; 0.39	0.16^‡^	0.04; 0.27
HbA1c (%)	1.77^†^	-0.84; 4.39	0.68	-1.41; 2.78
Insulin (U/L)	0.24^‡^	0.03; 0.45	0.23^‡^	0.06; 0.40
HOMA-IR	1.19^‡^	0.17; 2.21	1.07^‡^	0.25; 1.88
TG (mg/dL)	0.04^§^	0.02; 0.06	0.03^‡^	0.01; 0.04
TC (mg/dL)	-0.001	-0.03; 0.02		
LDL (mg/dL)	0.008	-0.02; 0.03		
HDL (mg/dL)	-0.18^§^	-0.27; -0.09	-0.06^‡^	-0.12; -0.002
BMI (kg/m^2^)	1.49^§^	1.28; 1.71	1.41^§^	1.20; 1.63
WC (cm)	0.68^§^	0.58; 0.79	0.58^§^	0.48; 0.69
**DBP**				
Glucose (mg/dL)	0.10^‡^	0.01; 0.19	0.08^‡^	0.002; 0.17
HbA1c (%)	1.09^†^	-0.56; 2.76	0.65	-0.77; 2.07
Insulin (U/L)	0.13^†^	-0.05; 0.31	0.09	-0.05; 0.24
HOMA-IR	0.62^†^	-0.26; 1.51	0.45	-0.25; 1.16
TG (mg/dL)	0.02^‡^	0.01; 0.03	0.01^‡^	0.005; 0.02
TC (mg/dL)	0.02^‡^	0.00; 0.03	0.02^‡^	0.007; 0.03
LDL (mg/dL)	0.01^‡^	0.00; 0.03	0.01^‡^	0.002; 0.03
HDL (mg/dL)	-0.01	-0.06; 0.02		
BMI (kg/m^2^)	0.57^§^	0.43; 0.72	0.56^§^	0.40; 0.72
WC (cm)	0.27^§^	0.20; 0.33	0.25^§^	0.18; 0.33

BMI: body mass index; CI: confidence interval; DBP: diastolic blood pressure; HbA1c: glycated hemoglobin; HDL: high-density lipoprotein; HOMA-IR: homeostatic model assessment for insulin resistance; LDL: low-density lipoprotein; SBP: systolic blood pressure; TG: triglycerides; TC: total cholesterol; WC: waist circumference.^†^p < 0.20;^‡^p < 0.05;^§^p < 0.001. * Analysis was adjusted for sex, age, sexual maturity stage, skin color or ethnicity, type of school, and presence or absence of obesity.

## Discussion

To our knowledge, this is the first study to investigate hypertension in school-aged adolescents in the Federal District. The estimated prevalence was similar to that found for the Brazilian regions of Midwest (8.7, 95% CI: 7.9; 9.6), North (8.4, 95% CI: 7.7; 9.2), Northeast (8.4, 95% CI: 7.6; 9.2), Southeast (9.8, 95% CI: 8.8; 11.0), and for the national sample of ERICA (9.6, 95% CI: 9.0; 10.3). It was lower than the estimated prevalence for the South (12.5, 95% CI: 11.0; 14.2) only.^7^ A high prevalence of alterations other biochemical and adiposity markers was described, and the reported associations may potentiate cardiovascular risk in this population.

Similar to the findings of ERICA,^7^ although most adolescents studied in urban areas, hypertension was more common in rural schools. An explanatory hypothesis is that rural environments often have limited access to health care services, which hinders the diagnosis and treatment of chronic diseases such as hypertension.^21^

The evaluation of glucose metabolism markers in adolescents showed that high levels in fasting blood glucose were less prevalent than those in other markers. However, the evaluation of blood glucose alone is insufficient to rule out metabolic changes because, at the onset of IR, blood glucose may remain within normal levels as a consequence of a possible hyperinsulinemia.^14^ Changes in HbA1c, which were more prevalent in these students, may be a better marker in the evaluation of glycemic control as they reflect blood glucose changes in the long term.^22^

High blood glucose levels favor hypertension through an increase in cardiac output caused by hyperosmolality induced by hyperglycemia.^23^ Increased blood glucose can also lead to an excessive generation of reactive oxygen species (ROS), which contributes to endothelial dysfunction.^24^ When prolonged, hyperglycemia can also contribute to the generation of advanced glycation end-products, which intensify oxidative stress by activating a proinflammatory cascade. This increases the expression of ROS and contributes to the inhibition or reduction of nitric oxide production, leading to peripheral vascular resistance by vasoconstriction.^24 , 25^

In addition, there was an increase in insulin and HOMA-IR levels. Andrade et al.^26^ suggest, however, that IR may be related to development during adolescence, involving hormonal and body composition changes in the early stages of puberty. They believed this could be reversed after the growth spurt, which does not explain the present results because most of the study population was in the final stage of puberty. Other studies have also reported higher insulin levels and HOMA-IR changes in adolescents with hypertension compared with other groups.^27 , 28^

Adolescents with hypertension also had higher BMI and WC, which is consistent with the findings of other studies.^29 , 30^ High adiposity contributes to hypertension, among other mechanisms, by favoring oxidative stress with the onset of a proinflammatory state, with increased expression of cytokines such as interleukin 6 (IL-6) and tumor necrosis factor-alpha.^31^ Inflammation is an important mediator both in the onset and in the maintenance of high blood pressure levels because it may cause vascular and renal damage.^32^ In addition, obese individuals may show a greater degree of sympathetic activation, with an increased renal production of norepinephrine and a consequent increase in renal tubular reabsorption of sodium.^33^ Sympathetic activation can be further stimulated by an excessive production of leptin, which is common in those with high adiposity.^34^ The presence of nonfunctional adipose tissue in obesity can also affect the renin-angiotensin system by increasing the circulating levels of angiotensin II and aldosterone; this causes hemodynamic changes that also contribute to increased blood pressure blood pressure,^35^ which explain the association observed in this study.

The presence of inflammation and endothelial dysfunction markers is also typical of dyslipidemias.^36^ Changes in lipid profile and presence of inflammatory cytokines such as IL-6 are related to increased arterial stiffness and thus to blood pressure, favoring the onset of hypertension and increasing the risk of developing cardiovascular diseases.^37^

The results of studies conducted in other regions of Brazil^29 , 38^ and the world^39 , 40^ corroborate the prevalences and associations found in the Federal District, and these findings reinforce the high frequency of cardiovascular risk factors at increasingly earlier ages.^41^ The unhealthy lifestyle of the Brazilian adolescent population, consisting especially of low food quality,^42 , 43^ sedentary behavior, high screen time,^44^ and emotional stress,^45^ potentiate the risks.

The interpretation of the results of this study is conditioned to some limitations of ERICA. The prevalence of hypertension may have been overestimated because blood pressure was measured on a single day.^12^ However, in large cross-sectional studies such as ERICA, this is a common limitation as increasing the number of visits implies greater financial and logistics investments. It is worth noting that the methodology adopted on the day of collection may have reduced this bias.^8^

Despite these limitations, ERICA had appropriate quality monitoring processes for fieldwork and great methodological rigor for statistical analysis. These steps contributed to the robustness of the study and the reliability of the results.

## Conclusion

The estimated prevalence of hypertension in Federal District school-aged adolescents was 8%, associated with metabolic and adiposity markers. These findings highlight the metabolic links that may be present in hypertension. Within this context, health promotion and disease prevention actions are crucial to avoid epidemiological background reported in the Federal District and to contribute to improve the population’s quality of life and lower the burden to the health care system.
